# Human threat circuits: Threats of pain, aggressive conspecific, and predator elicit distinct BOLD activations in the amygdala and hypothalamus

**DOI:** 10.3389/fpsyt.2022.1063238

**Published:** 2023-01-09

**Authors:** Teresa Bertram, Daniel Hoffmann Ayala, Maria Huber, Felix Brandl, Georg Starke, Christian Sorg, Satja Mulej Bratec

**Affiliations:** ^1^Department of Neuroradiology, Klinikum Rechts der Isar, Technical University of Munich, Munich, Germany; ^2^TUM-NIC Neuroimaging Center, Klinikum Rechts der Isar, Technical University of Munich, Munich, Germany; ^3^Department of Psychiatry and Psychotherapy, Klinikum Rechts der Isar, Technical University of Munich, Munich, Germany; ^4^Department of Neurosurgery, Klinikum Großhadern, Ludwig-Maximilians-University, Munich, Germany; ^5^College of Humanities, École Polytechnique Fédérale de Lausanne, Lausanne, Switzerland; ^6^Department of Psychology, Faculty of Arts, University of Maribor, Maribor, Slovenia

**Keywords:** human, threat responses, translational neuroscience, threat conditioning, threat types, fMRI, threat circuit

## Abstract

**Introduction:**

Threat processing, enabled by threat circuits, is supported by a remarkably conserved neural architecture across mammals. Threatening stimuli relevant for most species include the threat of being attacked by a predator or an aggressive conspecific and the threat of pain. Extensive studies in rodents have associated the threats of pain, predator attack and aggressive conspecific attack with distinct neural circuits in subregions of the amygdala, the hypothalamus and the periaqueductal gray. Bearing in mind the considerable conservation of both the anatomy of these regions and defensive behaviors across mammalian species, we hypothesized that distinct brain activity corresponding to the threats of pain, predator attack and aggressive conspecific attack would also exist in human subcortical brain regions.

**Methods:**

Forty healthy female subjects underwent fMRI scanning during aversive classical conditioning. In close analogy to rodent studies, threat stimuli consisted of painful electric shocks, a short video clip of an attacking bear and a short video clip of an attacking man. Threat processing was conceptualized as the expectation of the aversive stimulus during the presentation of the conditioned stimulus.

**Results:**

Our results demonstrate differential brain activations in the left and right amygdala as well as in the left hypothalamus for the threats of pain, predator attack and aggressive conspecific attack, for the first time showing distinct threat-related brain activity within the human subcortical brain. Specifically, the threat of pain showed an increase of activity in the left and right amygdala and the left hypothalamus compared to the threat of conspecific attack (pain > conspecific), and increased activity in the left amygdala compared to the threat of predator attack (pain > predator). Threat of conspecific attack revealed heightened activity in the right amygdala, both in comparison to threat of pain (conspecific > pain) and threat of predator attack (conspecific > predator). Finally, for the condition threat of predator attack we found increased activity in the bilateral amygdala and the hypothalamus when compared to threat of conspecific attack (predator > conspecific). No significant clusters were found for the contrast predator attack > pain.

**Conclusion:**

Results suggest that threat type-specific circuits identified in rodents might be conserved in the human brain.

## 1. Introduction

Processing threats is vital to an organism’s chance of survival. On the anatomical level, threat processing is associated with brain circuits that extend from subcortical circuits in the hypothalamus and periaqueductal gray (PAG), to the amygdala, striatum and hippocampus, and finally to cortical areas such as prefrontal and cingulate cortices (1–3). The involved subcortical structures, such as hypothalamus, PAG and amygdala, are known to be structurally highly conserved throughout the mammalian evolution (4–6). On the behavioral level, many responses to threat, called defensive responses, are highly consistent across mammals (7). In rats and mice, for example, unambiguous threat stimuli, such as a close predator, tend to result in escape, when an escape route is available, and freezing, when it is not (8). In contrast, if the threat stimulus is ambiguous or partial (e.g., the smell of a predator), the prototypical response is to orient to and investigate the stimulus (risk assessment) (9). Very similar behavioral patterns are observed in humans (10–14).

**FIGURE 1:**
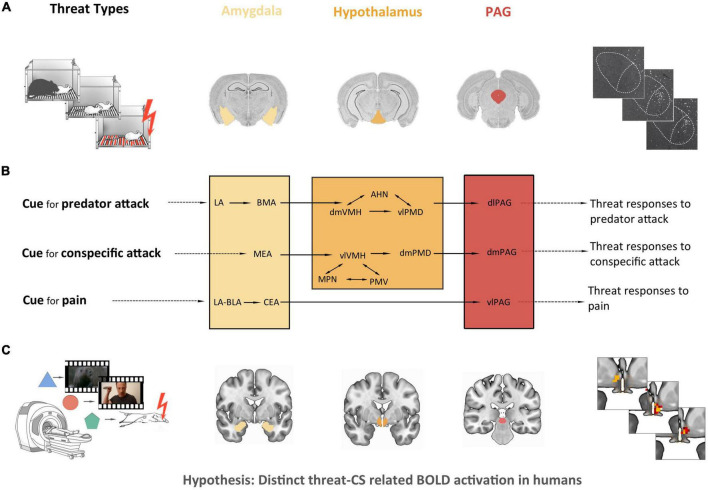
Study’s empirical and theoretical background and derived hypothesis. **(A)** Empirical background: a number of immunohistochemical, electrophysiological, and lesion studies in rodents have found that the threat of pain, predator attack and aggressive conspecific attack engage distinct neural circuits in subregions of the amygdala, the hypothalamus and the periaqueductal gray (PAG). From left to right: simplified illustration of the experimental setup for three distinct threat types, adapted from Canteras et al. (131); coronal histological slices of the three regions of interest, based on the Allen Mouse Brain Atlas (132); example of an immunohistochemical outcome (c-Fos-expressing cells in the ventromedial hypothalamus), based on Wang et al. (133). **(B)** Theoretical background: model of distinct threat circuits by Gross and Canteras (15): threat of pain recruits the basolateral amygdala (BLA), lateral amygdala (LA) and central amygdala (CEA) to generate defensive responses to pain via the ventrolateral PAG (vlPAG). Threat of predator attack involves LA and basomedial amygdala (BMA), the dorsomedial part of the ventromedial hypothalamus (dmVMH), the anterior hypothalamic nucleus (AHN), the ventrolateral part of the dorsal premamillary nucleus (vlPMD), and the dorsolateral PAG (dlPAG), triggering defensive responses to a predator attack. Threat of aggressive conspecific attack recruits the medial amygdala (MEA), the ventrolateral part of the VMH (vlVMH), the dorsomedial PMD (dmPMD), the medial preoptic nucleus (MPN), the ventral premammillary nucleus (PMV), and the dorsomedial PAG (dmPAG), resulting in defensive responses to a conspecific attack. Adapted from Gross and Canteras (15). **(C)** Hypothesis: given the considerable degree of mammalian evolutionary conservation of threat behavior and of the anatomy of the implied regions, the question arises of whether distinct neural pathways for different types of threat exist also in the human brain. The present fMRI study investigated the neural correlates of three analogous types of threat (threat of pain, threat of predator attack and threat of conspecific attack) in 40 healthy subjects with a conditioning paradigm, hypothesizing differential conditioned stimulus (CS)-related brain activation in the amygdala, the hypothalamus and the PAG.

Although the precise sources of threat vary among species, one can distinguish basic types of threatening stimuli that are relevant for most species [see (15)]. These include the threat of being attacked by an aggressive conspecific or a predator and the threat of pain, such as the threat of injuring one’s body. Remarkably, studies in rodents have associated different types of threatening stimuli with distinct subcortical pathways (16–23); for reviews see Gross and Canteras (15) and Silva et al. (24). They suggest that the threats of pain, predator attack and aggressive conspecific attack engage distinct neural circuits within the amygdala, the hypothalamus and the PAG (see Figure 1). For instance, threat of pain in rodents was shown to recruit preferentially the basolateral amygdala (BLA), lateral amygdala (LA) and central amygdala (CEA) to generate defensive responses to pain via the ventrolateral PAG (vlPAG). Threat of predator attack, on the other hand, was shown to involve mainly LA and basomedial amygdala (BMA), the dorsomedial part of the ventromedial hypothalamus (dmVMH), the anterior hypothalamic nucleus (AHN), the ventrolateral part of the dorsal premammillary nucleus (vlPMD) and the dorsolateral PAG (dlPAG), while the threat of aggressive conspecific attack in rodents was demonstrated to recruit the medial amygdala (MEA), the ventrolateral part of the VMH (vlVMH), the dorsomedial PMD (dmPMD), the medial preoptic nucleus (MPN), the ventral premammillary nucleus (PMV), and the dorsomedial PAG (dmPAG).

Not surprisingly, studies in humans hint at the involvement of these same brain structures in processing threat signals. fMRI studies have implicated the human amygdala in pain conditioning [reviewed by (25)] as well as in response to fearful conspecific faces [e.g., (26)]. Mobbs et al. (27) showed that as a virtual predator approached, brain activity shifted from the ventromedial prefrontal cortex to the PAG. A modification of the experiment showed an increase in PAG activity the closer a tarantula was placed to the foot of the subject lying in the scanner (28). In addition, electrically stimulating the dorsal PAG and the ventromedial hypothalamus elicits feelings of panic [reviewed in (29)]. Threat images, compared to merely negative images, have been found to evoke greater and earlier BOLD activations in the amygdala and PAG (30). A recent study examined humans with bilateral calcifications of the BLA and rats with BLA lesions and found evidence that the BLA is necessary for switching from passive defensive to active escape behavior in both species (31).

It is unclear, however, whether different threat types distinctively recruit these subcortical regions in the human brain. Given the considerable degree of anatomical conservation of the implicated regions (4–6) and the similarities in defensive behaviors across mammalian species, we aimed to investigate whether different types of threat would also differentially activate the human subcortical brain.

This issue is of great importance, because dysregulated threat processing and defensive responses characterize many neuropsychiatric disorders. In anxiety disorders, major depression, and chronic pain, for instance, attentional biases to threatening stimuli play an important role in the development and maintenance of the disorder (32–35). Drugs that are clinically effective against anxiety disorders in humans modulate defensive behavior in rodents (36–38). The benzodiazepine lorazepam, which has anxiolytic effects, has been shown to modulate the expression of avoidance behavior toward threatening stimuli in humans (39). Elucidating basic neural mechanisms that support adaptive threat processing and behavior is thus crucial to gaining a better understanding of disorders characterized by maladaptive responses to threat.

Translating research paradigms from animal to human studies and vice versa brings with it a number of methodological challenges, however. For instance, animal studies can investigate more ethologically valid threatening situations, whereas ethical considerations place restraints on the nature and intensity of aversive stimuli which can be used in human studies. Despite methodological disparities, a large body of work investigating threat conditioning in animals and humans has found a largely overlapping core neural network involved in conditioning and extinction (25, 40–42), suggesting that this experimental paradigm can detect meaningful and comparable underlying neural mechanisms across species.

To investigate the neural correlates of distinct threat types in the human brain, we used functional magnetic resonance imaging (fMRI) to measure blood-oxygenation-dependent (BOLD) activity and examined differential brain activations to three types of threat in 40 healthy females within a conditioning paradigm. Based on the principle of classical Pavlovian conditioning (43), threat conditioning is a widely used paradigm in translational neuroscience.

We specifically focused on threat processing (rather than defensive responses, which are more challenging to investigate in humans in a scanner setting), which was conceptualized as the expectation of the aversive, unconditioned stimulus (US) during the conditioned stimulus (CS) presentation. In analogy to the rodent studies the aversive stimuli in our study were painful electric shocks (“threat of pain”), a short video clip of an attacking bear (“threat of predator attack”) and a short video clip of an attacking aggressive man (“threat of conspecific attack”). Because of the different sensory modalities of the conditions threat of pain (electric shocks) and threat of predator/conspecific attack (audio-visual stimuli) we decided to focus the analysis on CS instead of US-related brain activity. This allowed for a comparison across conditions without confounding effects of different properties of the aversive stimuli.

We hypothesized that the conditions threat of pain, threat of predator attack and threat of conspecific attack would involve distinct CS-related BOLD activations within the amygdala, hypothalamus and PAG of healthy humans.

## 2. Materials and methods

### 2.1. Ethics statement

This study was approved by the ethics committee at the Technical University of Munich. All participants gave their informed consent and received monetary compensation for their participation.

### 2.2. Participants

Forty-five healthy females with a mean age of 23.6 years (*SD* = 4.1) took part in the experiment, carried out at the Technical University of Munich in Germany. All participants were right-handed native German speakers with normal or corrected-to-normal vision, reported no history of psychiatric or neurological disorders and no current intake of psychoactive medication. Data from five participants were excluded from analysis due to excessive head-movement during fMRI acquisition (*N* = 1), a high score for depressive symptoms [a score of 22 on the Beck Depression Inventory (44), *N* = 1], extreme sleepiness during the experiment detected by eye monitoring (*N* = 2) or technical error (*N* = 1). Only female subjects were included in the study due to previously reported sex differences in fear conditioning in humans and rodents [reviewed in (45)]. For instance, male rodents acquire CS-US associations faster than females and display freezing behaviors more frequently than females (46, 47). Similarly, in humans, larger conditioned responses during fear acquisition were found in men relative to women (48). Human fMRI studies on fear conditioning with painful stimuli have shown significantly greater BOLD-signal changes in the right amygdala, right rostral anterior cingulate (rACC) and dorsal anterior cingulate cortex (dACC) (49) and more insula activation (50) in women compared with men. In order to increase sample homogeneity (and thereby signal-to-noise ratio) and because of the higher occurrence of anxiety and stress-related disorders in females compared to males (51, 52), we chose only female subjects for the study.

### 2.3. Experimental design

**FIGURE 2:**
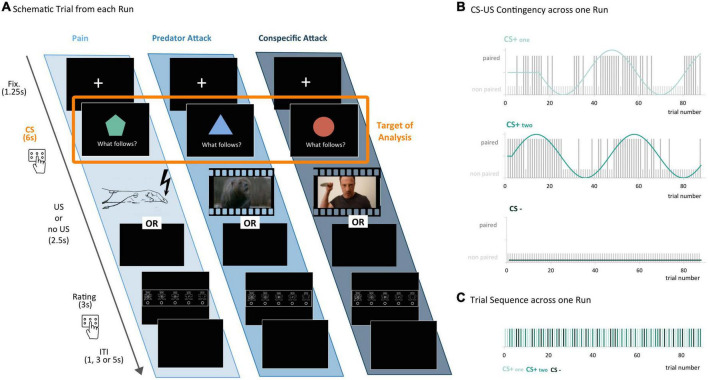
Experimental design – threat conditioning for three distinct threat types. **(A)** Schematic trial from each run: each trial began with a fixation cross, followed by a conditioned stimulus (CS) in the form of a simple geometric shape. During the first three seconds of CS presentation, participants were asked to predict whether they believed an unconditioned stimulus (US) would follow or not, by pressing a button. The US differed according to threat type condition: Pain – electric shocks applied to the participant’s wrist; predator – an audio-visual stimulus picturing a roaring bear jumping toward the viewer; conspecific – an audio-visual stimulus of an aggressive man coming toward the viewer with a knife in his hands. When no US followed, the screen remained black. Next, participants were asked to rate their arousal on the Self-Assessment Manikin (SAM) scale. Each trial ended with an inter-trial-interval (ITI) of varying length. **(B)** CS-US Contingency across one run: the CS–US contingency was modulated according to a sinusoidal function in order to avoid habituation effects. In each run, there were two reinforced CSs (CS+_one_ and CS+_two_, paired with the US in 50% of all cases, with phase-shifted contingency functions but followed by the same US), and one non-reinforced CS (CS–; never paired with a US, serving as a baseline in later analyses). **(C)** Trial sequence across one run: the experiment consisted of three runs (one for each threat type) with 88 trials each. Shown here is an exemplary sequence of CS+_one_, CS+_two_ and CS– trials in one run.

To investigate the neural correlates of different threat types in the human brain, three different types of aversive stimuli were presented within a conditioning paradigm with fluctuating conditioned–unconditioned stimulus (CS–US) contingencies (Figures 2A, B). The design was adapted from two previous studies, where it had been used for conditioning with painful stimuli (53) and aversive pictures (54). The conditioning paradigm was chosen since the focus on CS-related activity enabled the comparison of brain activity across different threat types. This was particularly relevant due to the different sensory modalities of the conditions threat of pain (electric shocks) and threat of predator/conspecific attack (audio-visual stimuli). Focusing the analysis on CS- instead of US-related brain activity allowed for a comparison across conditions without confounding effects of different properties of the aversive stimuli such as sensory modality or intensity.

The three types of US in the current study included painful stimuli in the form of electric shocks (for the condition threat of pain) as well as two audio-visual stimuli with a length of 2.5 s simulating an attack by a predator and by a conspecific (for the conditions threat of predator attack and threat of conspecific attack, respectively). In the predator attack condition, the US was a video showing a roaring bear leaping toward the viewer with a wide-open jaw. The video consisted of scenes from the horror movie Into the Grizzley Maze (55). In the conspecific attack condition, the video showed an aggressive young man with a knife in one hand, coming toward the viewer. The scene was shot with the help of a befriended actor and cinematographer. Both video clips were cut with the software iMovie (Version 10.0.7, Apple Inc.) and are available upon request. In each attack condition, the same video clip was repeated across trials, to increase comparison with the electric shock condition. The electric shocks were applied to the dorsum of the right wrist for a duration of 1 s, using a constant voltage stimulator (STM200, Biopac Systems, Goleta, CA, United States). In order to ensure that the level of perceived pain was comparable across participants, we performed a calibration procedure with each participant, following the example of previous studies on pain conditioning (56–58). Specifically, the current was first increased gradually to define the minimum current the participant was able to feel as well as their subjective maximum, defined as the highest tolerable pain. Within this range, a number of test shocks of random intensities were applied and the participants were asked to rate the intensity of each shock on a numerical rating scale from 0 (not painful at all) to 10 (highest tolerable pain). A sigmoid function was fitted to the acquired data points and finally a single individualized current corresponding to a rating of 8/10 according to that fit was chosen.

The experiment consisted of three runs, one per threat type, with 88 trials each. On each trial, participants had to predict whether a CS would be followed by the US or by a black screen (see Figure 2A). In each threat type condition (pain, predator, and conspecific) there was only one type of US (electric shocks, video of an attacking bear or video of an attacking conspecific, respectively), but three different CSs (CS+_one_, CS+_two_, and CS−, consisting of different simple geometric shapes (see Figure 2A). Two of the three CSs were associated with the US: CS+_one_ and CS+_two_ were reinforced CSs, which were paired with the US in 50% of all cases. The third CS (CS−) was a non-reinforced CS, which was never paired with the US and served as a baseline in later analyses (Figures 2B, C). The participant’s task was to correctly predict whether a US would follow a particular CS. To avoid habituation effects (53, 59), the CS–US contingency of CS+_one_ and CS+_two_ was modulated according to a sinusoidal function (Figure 2B). The order of the three runs and the colors and shapes of the CSs were counterbalanced across participants. At the end of each trial, participants were asked to rate their current arousal on the Self-Assessment Manikin (SAM) scale (60).

### 2.4. Experimental procedure

Participants were informed that the purpose of the study was the investigation of brain activity during emotional learning and that it would include aversive audio-visual stimuli and painful electric shocks. Informed consent was followed by a short training session, in which participants practiced the task on a laptop outside the scanner room until they could successfully predict the occurrence or non-occurrence of the US. An audio-visual stimulus picturing a passing streetcar exemplified the US in the training session. Inside the scanner, participants were first asked to assist with the calibration of the electric shocks. Afterward they completed the fMRI session, consisting of three runs, lasting 24 min each.

### 2.5. Behavioral measures

#### 2.5.1. Arousal ratings for the unconditioned stimuli

Participants rated their arousal at the end of each trial. To test whether the US successfully induced a threat response (i.e., an aversive emotional response) in each condition, we conducted a 2 × 3 ANOVA on SAM arousal scores with within-subjects factors US Presence (US Absent and US Present) and Threat Type (Pain, Predator, and Conspecific), as well as three *post hoc* 2 × 2 ANOVAs and *post hoc* paired *t*-tests. A significance level of α = 0.05 was set for all tests. Effect sizes were estimated as partial eta squared for ANOVAs and as Cohen’s *d* for *t*-tests.

#### 2.5.2. Effect of conditioning for each threat type

During CS presentation, participants had to predict via a button press whether they believed the US would follow or not. To make sure that the conditioning procedure was successful, in other words that participants expected the US more often after CS+ compared to CS− presentation, the proportion of “US present” predictions was calculated for each participant, separately for each threat type. A 2 × 3 ANOVA on the proportion of “US present” predictions with the factors CS Type (CS+ and CS−) and Threat Type (Pain, Predator, and Conspecific) as well as three *post hoc* 2 × 2 ANOVAs and *post hoc t*-tests were performed.

### 2.6. Physiological measures

Physiological noise induced by cardiac and respiratory cycles accounts for a considerable amount of variance in the BOLD signal, especially in the areas close to the brainstem, such as the ones our hypothesis focused on (61). To estimate the impact of physiological noise, we recorded respiratory and cardiac phase throughout the experiment and entered this data in a retrospective image correction (RETROICOR) noise model as implemented by the PhysIO toolbox (62). The resulting nuisance regressors were included in the General Linear Model (GLM) analysis. Respiratory data was recorded with an inductive belt placed around the ribcage. Cardiac phase was derived from a photoplethysmographic signal measured with a pulse-oxymeter fixated on the ring finger of the left hand. Both measures were recorded using the Biopac System MP150 together with the AcqKnowledge Software (Biopac Systems, Goleta, CA, United States).

### 2.7. Neuroimaging: Data acquisition and preprocessing

The MR imaging was performed with a 3 T Philips Ingenia scanner with a 32-channel head coil at the Technical University of Munich. T1-weighted anatomical images were acquired using a magnetization-prepared acquisition gradient echo sequence (MPRAGE) with a resolution of 0.67 mm × 0.67 mm × 0.70 mm. For the functional images we used interleaved multiband imaging with a factor of 2 and a contrast-gradient echo-planar T2*-weighted sequence (EPI) covering the whole brain (repetition time = 2,700 ms, echo time = 26 ms, flip angle = 90°, acquisition matrix = 96 × 94, 64 slices, slice thickness = 2 mm, no gap, in-plane resolution = 2 mm × 2 mm). Audio-visual stimuli were presented using Presentation software (Neurobehavioral Systems, Inc., Berkeley, CA, United States), which received trigger pulses from the scanner for synchronization with image acquisition. Visual information was projected on a screen at the head of the scanner, viewable through an adjustable mirror. Sound was transmitted via MR-compatible headphones.

Image processing and statistical analysis was completed with SPM 12 (Wellcome Trust, London, UK), running on MATLAB 2016b (The MathWorks Inc., Natick, MA, United States). After discarding the first two volumes, the functional images were slice time corrected, realigned to the first image of each run and unwarped. The participants’ structural images were co-registered to the functional images, segmented and then normalized to a standard T1 template in the Montreal Neurological Institute (MNI) space. These normalization parameters were applied to the functional images, which were then smoothed with a 4 mm full-width-at-half-maximum Gaussian filter.

The control of movement-related artifacts, including vessel-induced artifacts in the brainstem and physiological ‘noise’ such as heartbeat, were performed at the subject-specific first level data analysis, which was based on the GLM approach, as described below.

### 2.8. Neuroimaging: Data analysis

#### 2.8.1. First-level analysis

A first-level GLM was estimated for each subject using the following event-related regressors: hemodynamic response function (HRF)-convolved onsets of CS+_one_, CS+_two_, CS−, US-present_one_, US-present_two_, US-absent_one_, US-absent_two_, US-absent_minus_, and arousal rating scale. The GLM also included the following regressors of no interest: (1) parametric modulations of CS onset regressors, using participant’s trial-wise arousal ratings, to control for the additional variance related to arousal fluctuations over the course of the run; (2) the 1st order derivatives of the six movement regressors obtained during the realignment procedure (63); (3) 18 regressors accounting for cardiac and respiratory noise, derived from the RETROICOR (physiological noise) model with the default settings: a 3rd order cardiac model (six regressors, sine/cosine), 4th order respiratory model (eight regressors), and a 1st order interaction model (four terms); (4) motion censoring regressors: a temporal mask flagging volumes with >2 mm/degrees of head motion. Via the motion censoring regressors, volumes in which head motion exceeded a threshold of 2 mm translation and 2° rotation were withheld from GLM estimation, adapting the strategy known as “motion censoring” (63). If this threshold was exceeded in more than three instances per run, however, the subject was excluded from all analyses (*N* = 1).

#### 2.8.2. Second-level analysis

Subjects’ parameter estimate maps for CS-related activity were then entered into a second-level 2 × 3 factorial analysis with factors CS Type (CS+ and CS−) and Threat Type (Pain, Predator, and Conspecific). The aim of the analysis was threefold: (1) to confirm that the conditioning procedure was successful both at the region of interest level and the whole brain level – by investigating the main effect of CS Type, (2) to test our hypothesis of differential activity corresponding to the threats of pain, predator attack and conspecific attack in the amygdala, hypothalamus and PAG – by exploring the 3-way interaction effect between the factors CS Type and Threat Type and (3) to further investigate any differential threat type activity by examining *post hoc* comparisons between each threat type pair for the contrast CS+ vs. CS, for example pain (CS+vs. CS−) vs. conspecific (CS+vs. CS−).

With the exception of the additional whole-brain analysis for the main effect of CS Type, all analyses were restricted to the three regions of interest defined by the rodent threat pathway model (15): amygdala, hypothalamus, and PAG. The mask for the amygdala was based on the Jülich histological atlas as implemented in the SPM Anatomy toolbox (64), a widely used probabilistic cytoarchitectonic atlas, which allows for a differentiation into different nuclei of the amygdala, such as the basolateral and centromedial amygdala. Maps of the hypothalamus and PAG are not included in this atlas. For the hypothalamus, we therefore used the CIT168 atlas (65), a probabilistic *in vivo* anatomical atlas of subcortical nuclei, based on MRI data from 168 healthy adults. The PAG mask was derived from the connectivity-based segmentation by Ezra et al. (66), who used diffusion magnetic resonance imaging and probabilistic tractography to segment the human PAG, making it possible to investigate the four different columns of the PAG.

Statistical maps were corrected for multiple comparisons with the family-wise error rate (FWE) correction, using a threshold of *p* < 0.05, based on a height threshold of *p* < 0.005. For analyses within the regions of interest, FWE-correction was carried out at the peak level within a combined mask of amygdala, hypothalamus and PAG. For *post hoc* comparisons, which further explored the 3-way interaction result, FWE-correction was carried out within a mask of the significant clusters from the interaction result and was considered significant if below the *p*-value of 0.0083 (0.05/6, considering the 6 *post hoc* comparisons). For the whole brain analysis, FWE-correction was implemented at the cluster level, with the whole-brain as the volume of interest. Results are reported in Montreal Neurological Institute space.

### 2.9. Control analysis for habituation effects

Since a differential habituation to the US across the different conditions could represent a potential confound, we carried out a control analysis to investigate habituation responses to the three threat stimuli. At the first level, we substituted regressors for CS1, CS2, and CS3 correspondingly with three nearly equally long regressors representing the respective 1st, 2nd, and 3rd third of the run. All other design parameters and regressors were held equal to the main analysis. Comparing the first and last section of a task to model habituation is a practice repeatedly used in previous research (67–69). We computed contrast maps comparing CSplus (1st third) > CSplus (3rd third), which then entered a second-level analysis, where a Factorial 1 × 3 ANOVA with the factor Threat Type (Pain, Predator, and Conspecific) was performed and the interaction effect with *F*-Tests as a measure of differential habituation between experimental modalities was examined. Statistical maps were corrected for multiple comparisons with FWE-correction at the peak level, using a threshold of *p* < 0.05, based on a height threshold of *p* < 0.005, within the combined mask of amygdala, hypothalamus and PAG.

## 3. Results

### 3.1. Behavioral results

#### 3.1.1. Arousal ratings for the unconditioned stimuli

**FIGURE 3:**
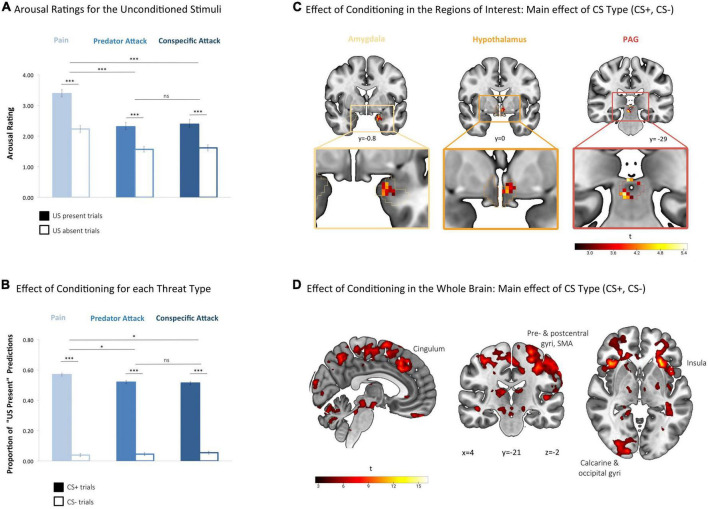
Threat conditioning. **(A)** Arousal ratings for the unconditioned stimuli. In each condition, trial-wise arousal ratings were significantly higher after the appearance of the unconditioned stimulus (US present) than the black screen (US absent), confirming that the stimuli were perceived as arousing by the participants. Arousal ratings were higher in the pain condition compared to the conditions predator attack and conspecific attack. Arousal was assessed with the Self-Assessment Manikin (SAM) scale and scores ranged from 1 (least aroused SAM) to 5 (most aroused SAM). Analysis based on a 2 × 3 ANOVA on arousal scores with within-subjects factors US Presence (US Absent and US Present) and Threat Type (Pain, Predator, and Conspecific) ^***^p < 0.001, based on *post hoc* two-tailed paired *t*-tests for each threat type (US present vs. US absent) and on the main effects of threat type in *post hoc* 2 × 2 ANOVAs for pain vs. conspecific attack and pain vs. predator attack. *ns*, not significant. Error bars represent standard error of the mean (SEM). **(B)** Effect of conditioning for each threat type. Participants expected the US significantly more often after CS+ compared to CS– presentation, suggesting that the conditioning procedure was successful at the behavioral level. In the pain condition participants expected the US slightly more often than in the other two conditions. Analysis based on a 2 × 3 ANOVA on the proportion of “US present” predictions with the factors CS Type (CS+, CS–) and Threat Type (Pain, Predator, and Conspecific). *p < 0.05, based on the main effects of threat type in *post hoc* 2 × 2 ANOVAs for pain vs. conspecific and pain vs. predator. ^***^p < 0.001, based on *post hoc* two-tailed paired *t*-tests for each threat type (CS+ vs. CS–). ns, not significant. Error bars represent standard error of the mean (SEM). **(C)** Effect of conditioning in the regions of interest: main effect of CS type (CS+, CS–). Significant main effects of CS Type were found in all three regions of interest, indicating that the conditioning procedure was successful in the amygdala, hypothalamus, and PAG. Based on a second-level 2 × 3 factorial analysis with factors CS Type (CS+, CS–) and Threat Type (Pain, Predator, and Conspecific). FWE-corrected (*p* < 0.05) at the peak level within amygdala, hypothalamus and PAG, based on a height threshold of *p* < 0.005. **(D)** Effect of conditioning in the whole brain: main effect of CS type (CS+, CS–). At the whole brain level, regions with significant main effects of CS Type coincide with the large-scale network consistently identified across fear conditioning studies, including the anterior insula, pre- and postcentral gyrus, SMA (supplementary motor area) and anterior cingulate gyrus. Based on a second-level 2 × 3 factorial analysis with factors CS Type (CS+, CS–) and Threat Type (Pain, Predator, and Conspecific). Whole-brain FWE-corrected (*p* < 0.05) at the cluster level (extent threshold of 144 voxels), based on a height threshold of *p* < 0.005.

To validate that the stimuli used in the experiment elicited a reliable threat response, we tested whether the US successfully induced increased arousal ratings in each threat condition. A 2 × 3 ANOVA on arousal scores with the factors US Presence (US Absent and US Present) and Threat Type (Pain, Predator, and Conspecific) was conducted. This revealed a significant main effect of US Presence (*F*[1,38] = 145.43, *p* < 0.001, ${\text{η}}_{\text{p}}^{2}$ = 0.79) with participants rating US present trials as more arousing than US absent trials in all threat conditions combined (Figure 3A). *Post hoc t*-tests revealed consistently higher self-reported arousal for US Presence for each of the three threat types (Pain: *t*[39] = 12.34, *p* < 0.001, *d* = 1.16; Predator: *t*[39] = 8.67, *p* < 0.001, *d* = 0.90; Conspecific: *t*[39] = 7.68, *p* < 0.001, *d* = 0.82).

The ANOVA also revealed a significant main effect of Threat Type on arousal ratings (*F*[1.33,50.56] = 61.61, *p* < 0.001, ${\text{η}}_{\text{p}}^{2}$ = 0.619) as well as a significant interaction effect between Threat Type and US Presence (*F*[1.49,56.64] = 13.03, *p* = 0.001, ${\text{η}}_{\text{p}}^{2}$ = 0.25), after Greenhouse–Geisser correction. *Post hoc* tests in the forms of 2 × 2 ANOVAs showed that the main and interaction effects remained significant when either of the two threat of attack conditions were taken out of the ANOVA (main effect of Threat Type [Pain, Predator]: *F*[1,39] = 85.72, *p* < 0.001, ${\text{η}}_{\text{p}}^{2}$ = 0.69; main effect of Threat Type [Pain, Conspecific]: *F*[1,38] = 57.56, *p* < 0.001, ${\text{η}}_{\text{p}}^{2}$ = 0.60), but not when the threat of pain condition was excluded (main effect of Threat Type [Predator, Conspecific]: *F*[1,38] = 2.09, *p* = 0.56, ${\text{η}}_{\text{p}}^{2}$ = 0.05). The US in the pain condition was thus perceived as more arousing by the participants (see Figure 3A). All in all, these findings confirm that all threat types were arousing to the participants, with painful stimuli being more arousing than the other two stimuli.

#### 3.1.2. Effect of conditioning for each threat type

To confirm that the conditioning procedure was successful on the behavioral level, we examined whether participants expected the occurrence of the US more often during CS+ than during CS− trials. ANOVA on the proportion of “US present” predictions revealed a significant main effect of CS Type (CS+, CS−) (*F*[1,39] = 1139.01, *p* < 0.001, ${\text{η}}_{\text{p}}^{2}$ = 0.97), with participants predicting the occurrence of the US significantly more often during CS+ than during CS− trials across conditions (Figure 3B). *Post hoc t*-tests confirmed that this was true for each of the three threat types (Pain: *t*[39] = 32.29, *p* < 0.001, *d* = 7.72; Predator: *t*[39] = 19.50, *p* < 0.001, *d* = 5.08; Conspecific: *t*[39] = 24.70, *p* < 0.001, *d* = 5.83). During CS presentation, participants were thus expecting the US to appear significantly more often during CS+ than during CS− trials, validating that the conditioning procedure was successful on the behavioral level. The analysis also revealed a small but significant main effect of Threat Type (*F*[2,78] = 4.109, *p* = 0.020, ${\text{η}}_{\text{p}}^{2}$ = 0.095) and an interaction effect between Threat Type and CS Type (*F*[2,78] = 4.895, *p* = 0.010, ${\text{η}}_{\text{p}}^{2}$ = 0.112). *Post hoc* tests in the forms of 2 × 2 ANOVAs showed that the main and interaction effects remained significant when either of the two threat of attack conditions were taken out of the ANOVA (main effect of Threat Type [Pain, Predator]: *F*[1,39] = 5.85, *p* = 0.020, ${\text{η}}_{\text{p}}^{2}$ = 0.130; main effect of Threat Type [Pain, Conspecific]: *F*[1,39] = 4.87, *p* = 0.033, ${\text{η}}_{\text{p}}^{2}$ = 0.111), but not when the threat of pain condition was excluded (main effect of Threat Type [Predator, Conspecific]: *F*[1,39] = 0.087, *p* = 0.769, ${\text{η}}_{\text{p}}^{2}$ = 0.002). Participants thus expected the US slightly more often in the pain condition, compared to the conditions predator attack and conspecific attack.

### 3.2. Neuroimaging results

#### 3.2.1. Effect of conditioning in the regions of interest: Main effect of CS type (CS+, CS−)

To verify that conditioning had occurred in our regions of interest, we explored brain activity corresponding to the main effect of CS Type (CS+, CS−) in the amygdala, hypothalamus and PAG. Significant effects of the factor CS Type were found in all three regions (see Table 1 and Figure 3C), indicating differential brain activity corresponding to the threatening CS+ (i.e., the expectation of the aversive stimulus) versus the non-threatening CS− (i.e., the expectation of a black screen) and thus suggesting an effect of the conditioning procedure on neural activity in our regions of interest.

**TABLE 1:** Effect of conditioning: main effect of CS type (CS+, CS−).

		Peak MNI coordinates				
Region	Cluster size	*x*-	*y*-	*z*-	Peak *z*	*p*(FWE-corr)
**(A) Regions of interest**						
Hypothalamus (R)	10	4	−12	−12	5.01	0.000
PAG	18	4	−28	−10	4.95	0.000
Hypothalamus (L)	16	−6	−8	−10	4.56	0.002
PAG	11	2	−30	−2	4.46	0.003
Hypothalamus (L)	17	−6	−2	−8	3.94	0.024
Basolateral amygdala (L)	19	−20	0	−20	3.78	0.042
**(B) Whole brain**						
Insula (L), middle frontal gyrus (L), inferior frontal gyrus (L)	2448	−28	26	−2	>8	0.000
Insula (R), middle frontal gyrus (R), anterior cingulum (R)	1673	34	24	−6	>8	0.000
Postcentral gyrus (L), precentral gyrus (L), supplementary motor area (R)	10339	−10	−10	60	>8	0.000
Middle frontal gyrus (R), inferior frontal gyrus (R)	946	38	24	26	7.78	0.000
Middle cingulum (R), superior frontal gyrus (R), anterior cingulum (L)	422	4	24	42	7.75	0.000
Cerebellum (R)	202	8	−64	−54	7.61	0.014
Cerebellum (L), cerebellum (R)	2444	−34	−58	−30	7.18	0.000
Supramarginal gyrus (R), superior temporal gyrus (R), angular gyrus (R)	273	62	−48	24	7.13	0.002
Middle temporal gyrus (R), superior temporal gyrus (R), inferior temporal gyrus (R)	201	46	−26	−6	6.86	0.014
Calcarine fissure and surrounding cortex (R), inferior occipital gyrus (R), lingual gyrus (R)	961	16	−96	−2	6.85	0.000
Cerebellum (L), cerebellum (R)	272	−8	−60	−52	6.69	0.002
Cuneus (R), cuneus (L), calcarine fissure and surrounding cortex (L)	192	6	−88	20	6.45	0.019
Thalamus (R), thalamus (L), pallidum (L)	1190	20	−28	8	6.42	0.000
Lingual gyrus (R), parahippocampal gyrus (R), calcarine fissure and surrounding cortex (R)	247	24	−60	0	5.28	0.004

(A) Regions of Interest: FWE-corrected (*p* < 0.05) at the peak level within amygdala, hypothalamus and PAG, based on a height threshold of *p* < 0.005. (B) Whole Brain: whole-brain FWE-corrected (*p* < 0.05) at the cluster level (extent threshold of 144 voxels), based on a height threshold of *p* < 0.005. Anatomical regions were identified with the Automated Anatomical Labeling (AAL) atlas; shown are the top three regions for each cluster (with the highest number of voxels).

#### 3.2.2. Effect of conditioning in the whole brain: Main effect of CS type (CS+, CS−)

We additionally examined the main effect of CS Type at the whole-brain level, to test whether the cortical activations would coincide with the regions typically associated with the anticipation of painful and audio-visual conditioned stimuli, see Fullana et al. (70) for a meta-analysis. Significant main effects of CS Type (CS+, CS−) were found in clusters extending over the left and right anterior and ventral insula as well as frontal operculum, pre- and postcentral gyrus, supplementary motor area, paracingulate and anterior cingulate gyrus, cerebellum, thalamus and occipital pole (see Table 1 and Figure 3D). These findings coincide well with the large-scale network consistently identified across threat conditioning studies (70), supporting the presence of a conditioning effect in our paradigm.

#### 3.2.3. Differential threat-related activity among threat types: Interaction between CS type (CS+, CS−) and threat type (pain, predator, and conspecific)

**FIGURE 4:**
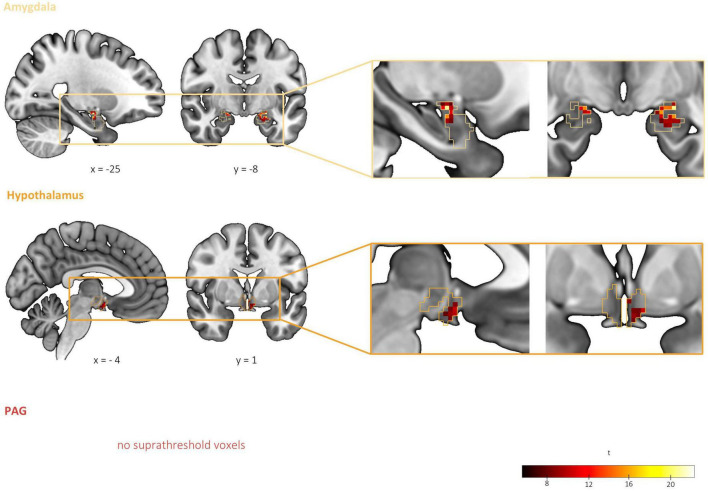
Differential threat-related activity among threat types: interaction between CS type (CS+, CS) and threat type (Pain, Predator, and Conspecific). Significant interaction effects were found in the left and right centromedial and basolateral amygdala as well as in the left hypothalamus, supporting our hypothesis of differential activation corresponding to the threats of pain, predator attack and conspecific attack. No significant clusters were detected within the PAG. Based on a second-level 2 × 3 factorial analysis with factors CS type (CS+, CS–) and threat type (Pain, Predator, and Conspecific). Height threshold of *p* < 0.005, FWE-corrected (*p* < 0.05) at the peak level within amygdala, hypothalamus and PAG.

To test our hypothesis of differential activity corresponding to the threats of pain, predator attack and conspecific attack in the amygdala, hypothalamus and PAG, we explored brain activations for the interaction effect of CS Type and Threat Type in these three regions. Significant interaction effects were found in the left and right CMA and BLA, as well as in the left hypothalamus (see Table 2 and Figure 4). No significant clusters were detected within the PAG. Results thus support our hypothesis of differential brain activations among the threats of pain, predator attack and conspecific attack within the amygdala and hypothalamus, but not the PAG.

**TABLE 2:** Differential threat-related activity.

		Peak MNI coordinates				
Region	Cluster size	*x*-	*y*-	*z*-	Peak *z*	*p*(FWE-corr)
**(A) 2 × 3 interaction between CS type (CS+, CS−) and threat type (pain, predator, and conspecific)**						
Basolateral amygdala (R)	17	30	2	−32	5.45	0.000
Centromedial and basolateral amygdala (L)	36	−24	−8	−14	4.99	0.000
Hypothalamus (L)	25	−6	−4	−15	4.48	0.004
Centromedial amygdala (R)	7	18	−8	−16	4.08	0.017
**(B) *Post hoc* comparisons between threat type pairs for the contrast CS+ > CS−**						
**Pain (CS+ > CS−) > conspecific (CS+ > CS−)**						
Centromedial and basolateral amygdala (L)	31	−16	−10	−16	5.01	0.000
Hypothalamus (L)	22	−2	2	−8	4.16	0.001
Centromedial amygdala (R)	6	18	−8	−14	4.61	0.000
**Pain (CS+ > CS−) < conspecific (CS+ > CS−)**						
Basolateral amygdala (R)	17	30	2	−32	5.77	0.000
**Pain (CS+ > CS−) > predator (CS+ > CS−)**						
Centromedial amygdala (L)	10	−24	−8	−14	4.83	0.000
**Pain (CS+ > CS−) < predator (CS+ > CS−)**						
**No suprathreshold voxels**						
**Conspecific (CS+ > CS−) > predator (CS+ > CS−)**						
Basolateral amygdala (L)	11	30	0	−32	4.49	0.000
**Conspecific (CS+ > CS−) < predator (CS+ > CS−)**						
Hypothalamus (L)	7	−6	−4	−14	4.95	0.000
Basolateral amygdala (L)	19	−28	−6	−20	4.12	0.001
Centromedial amygdala (R)	3	24	−12	−14	3.84	0.004

(A) Among threat types: interaction between CS type (CS+, CS−) and threat type (pain, predator, and conspecific) and (B) between threat type pairs: *post hoc* comparisons, FWE-corrected (*p* < 0.0083, Bonferroni-corrected for 6 *post hoc* tests) at the peak level within a mask of the significant voxels from the interaction result in amygdala and hypothalamus, based on a height threshold of *p* < 0.005. Subregions of the amygdala were identified with the Jülich histological atlas.

#### 3.2.4. Differential threat-related activity between threat type pairs: *Post hoc* comparisons

The condition threat of pain showed a relative increase of activity in the left BLA and CMA as well as the left hypothalamus compared to threat of conspecific attack (pain[CS+ > CS−] > conspecific[CS+ > CS−]), and increased activity in the left CMA compared to predator attack (pain[CS+ > CS−] > predator[CS+ > CS−]). Threat of conspecific attack, on the other hand, revealed heightened activity in the right BLA both in comparison to pain (conspecific[CS+ > CS−] > pain[CS+ > CS−]) and predator attack (conspecific[CS+ > CS−] > predator[CS+ > CS−]). Finally, for the condition threat of predator attack we found increased activity in the amygdala (left BLA, right CMA) and the hypothalamus when compared to conspecific attack (predator[CS+ > CS−] > conspecific[CS+ > CS−]). No significant clusters were found for the contrast predator (CS+ > CS−) > pain (CS+ > CS−). Looking at the three threat type pairs, results demonstrate significant differences between all three threat type pairs in the amygdala, and differences between two out of the three threat type pairs (pain vs. conspecific and predator vs. conspecific) in the hypothalamus. Consequently, the interaction effect was likely not driven by any one threat type, quite the opposite – we found a distinct pattern of activity for each threat type.

#### 3.2.5. Control analysis for habituation effects

To examine whether differential habituation to the US across the different conditions may have influenced our results, we performed a control analysis on habituation responses to the three threat stimuli. Small-volume-corrected results yielded no suprathreshold activations for the interaction effect of the threat modalities (threat of pain, threat of predator attack, and threat of conspecific attack). Thus, we could not find evidence for a confounding effect of habituation on differential threat-related activity in any of our regions of interest (amygdala, hypothalamus, and PAG).

## 4. Discussion

Inspired by findings in rodents, we tested the hypothesis of distinct threat type-dependent neural activity in the human amygdala, hypothalamus and PAG of healthy females using simultaneous fMRI and threat conditioning. We found differential CS-related brain activity for the threat of pain, predator and conspecific in the amygdala and hypothalamus. To the best of our knowledge, these findings represent first direct evidence of distinct threat type-dependent brain activity within the human subcortical brain, suggesting that rodent threat type-specific circuits might be conserved in the human brain.

### 4.1. Confirmation of conditioning success

In order to test the validity of our approach, the data were first examined for evidence that the conditioning procedure was successful. At the behavioral level, it was shown that the US of all three threat types elicited a significant aversive response and that the subjects expected the occurrence of the US significantly more often during CS+ than during CS− trials, indicating successful conditioning (Figures 3A, B). At the neural level, evidence for successful conditioning was found in the significant main effect of the factor CS-type (CS+, CS−) in all three region of interest (Figure 3C and Table 1A). In addition, the main effect of the factor CS-type (CS+, CS−) was examined at the whole brain level (Figure 3D and Table 1B). Significant clusters were found extending over the left and right anterior and ventral insula as well as the frontal operculum, pre- and postcentral gyrus, supplementary motor area, paracingulate and anterior cingulate gyrus, cerebellum, thalamus and occipital pole. These areas coincide well with the network that has been repeatedly identified in studies of conditioning with aversive (mainly painful) stimuli (70), providing additional evidence that conditioning was successful in the present study.

### 4.2. Amygdala

**FIGURE 5:**
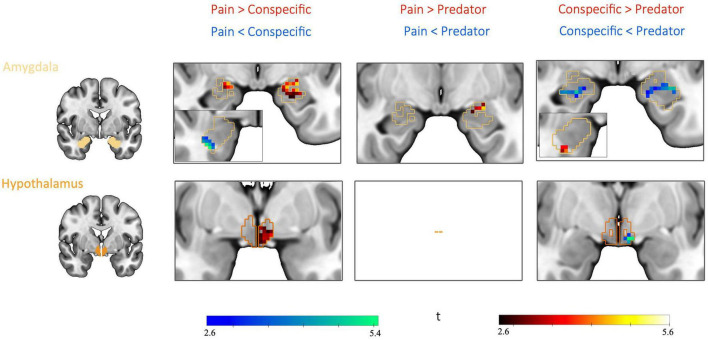
Differential threat-related activity between threat type pairs: *post hoc* comparisons. Pairwise contrasts revealed significant differences between all three threat type pairs in the amygdala as well as differences between two threat type pairs (pain vs. conspecific and predator vs. conspecific) in the hypothalamus. Based on *post hoc* comparisons between each threat type pair for the contrast CS+ > CS– (for example, pain[CS+ > CS–] vs. conspecific[CS+ > CS–]). FWE-corrected (*p* < 0.0083, Bonferroni-corrected for 6 *post hoc* tests) at the peak level within a mask of the significant voxels from the interaction result in amygdala and hypothalamus, based on a height threshold of *p* < 0.005.

We found differential CS-related activity between all the three threat types in the amygdala (Table 2 and Figures 4, 5). This finding is in line with a number of neural activity mapping and lesion studies in rodents, which have shown that different types of threatening stimuli depend on distinct subnuclei of the amygdala (16, 21, 71). The amygdala is a heterogeneous complex of several nuclei and plays an important role in the appraisal of the biological (i.e., basic motivational) relevance of a stimulus (72). The structural organization of the amygdala shows strong parallels across mammalian evolution (5, 73). In the context of threat, it has been proposed that the amygdala acts a site of integration, collecting threat-related afferents from different sensory modalities and channeling them along distinct downstream targets to produce contextually appropriate behaviors (74, 75).

The rodent threat circuitries are segregated at the level of amygdala outputs, with the predator cue circuit depending on outputs from the BMA, the conspecific attack cue circuit depending on outputs from the MEA and the pain cue responsive circuit depending on the BLA, LA, and CEA (15).

In humans, lesion studies have shown deficits in the recognition of threatening stimuli such as fearful facial expressions after removal of the amygdala in epilepsy patients or after calcification of the amygdala as a consequence of the Urbach–Wiethe disease (76–78). A large number of fMRI studies have implicated the human amygdala in fear conditioning with painful stimuli [reviewed by (25)] as well as in response to fearful faces of conspecifics [e.g., (26)]. Kveraga et al. (30) compared responses to threatening pictures to those of merely negative images and showed that the threat images triggered larger and earlier BOLD activations in the amygdala. Thus, while there is ample evidence for a role of the amygdala in processing threat in humans, it remains unknown whether different types of threat differentially recruit the amygdala. Although our data does not allow any inferences to be made at the level of individual nuclei, our findings suggest that a spatial segregation of circuits responsive to the threat of pain, predator attack and conspecific attack exists in the human amygdala (Figure 5).

### 4.3. Hypothalamus

In the hypothalamus, we found significant differential CS-related activity between the threat types predators and conspecifics as well as between pain and conspecifics (Table 2 and Figures 4, 5). The hypothalamus, an evolutionarily highly conserved region (6) at the base of the diencephalon, has been placed downstream of the amygdala in models of threat processing, controlling processes such as sympathetic outflow and the suppression of threat irrelevant behaviors, such as eating, drinking, and sexual behaviors (15).

The association of the hypothalamus with threat processing and defensive responses dates back to stimulation studies from the 1960s, in which it was discovered that stimulation of the hypothalamus in cats elicited “sham rage” responses (79). Lin et al. (80) applied optogenetic techniques showing that stimulation of the ventromedial hypothalamus in rats resulted in an attack on conspecifics as well as on inanimate objects. Case reports of humans following deep brain stimulation, specifically of patients with severe depression (81), chronic cluster headaches (82), and obsessive-compulsive disorder (83), describe strong feelings of anxiety and panic, as well as physical symptoms of hyperventilation, shortness of breath, and increased blood pressure after the electrical stimulation of the hypothalamus [summarized in (29)]. An activation of the hypothalamus has also been repeatedly reported in human fMRI studies with threatening stimuli (70, 84).

Our finding of distinct CS-related activity between the threat types predator and conspecific attack in the hypothalamus is in keeping with the rodent literature, which has identified sub-circuits within the medial hypothalamus that are preferentially recruited by predator-associated cue processing and by conspecific-associated cue processing, respectively (Figure 1) (16, 19, 85). Unlike the model of Gross and Canteras (15), which does not implicate the hypothalamus in the processing of pain related cues, we also found differential CS-related activations between the threat types pain and conspecific attack. While this is in line with more recent research, which implicates the medial hypothalamus in pain-related avoidance behavior (86, 87), further research is needed to corroborate our finding in the human brain.

### 4.4. PAG

Our results did not show differential threat-related activity among threat types in the PAG, despite significant conditioning effects in this region (Table 1 and Figure 3C).

The PAG forms a part of the ventricular gray matter and surrounds the midbrain aqueduct. It is commonly divided into four columns: the lateral PAG (lPAG), vlPAG, dlPAG, and dmPAG (88, 89). In addition to a role in pain perception and modulation (90, 91) and the regulation of respiratory and cardiovascular processes (92, 93), the PAG is considered an effector structure of defensive responses (15, 94, 95). Lesions in the dPAG attenuate responses such as risk assessment, flight or freezing in rats (20, 96), while electrical, pharmacological, or optogenetic stimulation of the PAG elicit defensive responses in rats, cats, and mice (97–102). Different columns of the PAG are thought to mediate distinct responses to different threatening stimuli (18, 103). More specifically, the threat of pain was preferentially associated with activation of the vlPAG, the threat of a predator with the dlPAG, and the threat of an aggressive conspecific with the dlPAG (15).

In contrast to the rodent literature, our study did not find differential PAG activations for the three threat conditions. Studies in humans have implicated the PAG in the processing of threatening stimuli. In a study with 12 patients with chronic pain syndromes, electrical stimulation of the dPAG induced strong feelings of panic (104). The aforementioned fMRI study, which compared responses to images of actual threat with those to merely negative images, found greater and earlier BOLD activation in the PAG in response to threat images compared with negative images (30). Mobbs et al. (27) showed that with the approach of a virtual predator, brain activity shifted from the ventromedial prefrontal cortex to the PAG. The PAG is thought of as an “output” structure in the context of threat, mediating distinct defensive responses (18, 103). As our subjects were lying in the scanner in all three threat conditions and were thus limited in terms of their responses and escape possibilities, we do not necessarily expect different “efferent” limbs of the treat circuit to be activated, compared to the rodent studies where distinct behavioral responses to different threats were observed in more ecologically valid settings. It seems that the experimental setting and/or the nature of aversive stimuli in our study was not sufficient to mediate distinct PAG-mediated responses for different threat types.

### 4.5. Control analysis

To investigate whether differential habituation to the US across the different conditions could explain some of the differential activations, we carried out a control analysis to investigate habituation responses to the three threat stimuli. We found no significant differential habituation effects across threat types in the regions of interest (amygdala, hypothalamus, and PAG), demonstrating that habituation effects did not impact our results.

### 4.6. Clinical relevance

Together, our findings point to the existence of distinct neural correlates of being threatened by pain, predator attack and conspecific attack in the human amygdala and hypothalamus.

Elucidating neural mechanisms that support adaptive threat processing and responding is relevant for our understanding of maladaptive threat processing, especially as many neuropsychiatric disorders are thought to be characterized by dysregulated processing and/or responding to threats (34, 105). Evolutionary theories of depression, for example, relate depressive symptoms to a defensive strategy known as ‘arrested flight’ (106), in which experiences of defeat and entrapment in a threatening environment are met with protective behaviors such as social withdrawal (107, 108). The development of depression has also been linked to the chronic exposure to environmental stressors. One of the major biological stress response systems in humans is the hypothalamic–pituitary–adrenal (HPA) axis, which has been shown to be altered in many individuals with depression through an impaired negative feedback mechanism leading to increased concentrations of cortisol (109, 110). Neurotransmitters implicated in the pathogenesis of depression, such as serotonin, are known to be involved in the regulation of the HPA axis (111). Furthermore, many depressive symptoms, such as disturbances in appetite, sleep or sex drive, point to disturbances in the hypothalamic function. In anxiety disorders, hyper-vigilance to threatening cues and responses in the absence of appropriate threats are thought to play a large role in the pathology, with consequent avoidance behaviors contributing to the maintenance of the conditions (112, 113). A meta-analysis of 172 studies showed that threat-related attentional biases were reliably found with different experimental paradigms across children and adults with different anxiety disorders as well as high-anxious non-clinical populations (114). Hyperactive amygdala responses to negative stimuli were found in a meta-analysis of positron emission tomography (PET) and fMRI studies in individuals with social anxiety disorder, specific phobia and posttraumatic stress disorder (115). The PAG, on the other hand, has been implicated in the pathophysiology of panic disorder, since electrical stimulation of the PAG has been shown to induce panic-like symptoms (104, 116). Interestingly, increased gray matter volume in the midbrain has been found in patients with panic disorder (117) and was shown to correlate with the severity of the disorder (118). A third example is pain, driven by actual or potential injury that leads to the avoidance of future threats and/or protective behaviors, which can become maladaptive in the form of chronic pain (34).

Interestingly, drugs, which show anxiolytic and antidepressant properties in humans, modulate defensive behavior in rodents (36–38, 119). For instance, the repeated administration of the antidepressants imipramine and fluoxetine (37) as well as the benzodiazepine alprazolam (119) attenuated defensive responses, such as defensive attack, in mice confronted with rats. Similarly, in humans, the benzodiazepine lorazepam has been shown to modulate the expression of avoidance behavior toward threatening stimuli (39).

In light of these considerations, it will be interesting to examine whether and how the subcortical neural architecture supporting processing and/or responses to distinct types of threatening stimuli is affected in patients with major depression, anxiety disorders and chronic pain. A better understanding of the pathomechanisms of threat related disorders will hopefully improve therapeutic approaches in the future.

### 4.7. Limitations

A number of limitations are worth noting. In comparison to the rodent studies that motivated this work, translation to human experiments involves inherent restraints. For instance, we can only investigate experimental analogs to experiences of threat, such as using film clips for predator and conspecific attacks. We are moreover confined to the spatial and temporal resolution of fMRI, not allowing inferences at the level of nuclei or individual neurons.

Furthermore, the BOLD signal may be driven by excitatory and/or inhibitory neural activity prohibiting differentiation between neural activations or deactivations (120), particularly so in our regions of interest where the nature of the neurovascular coupling is less well studied than in the cortex (121) and where noise induced by cardiac and respiratory phases plays a larger role due to brainstem proximity (61). We accounted for the impact of physiological noise on our data as far as possible by including cardiac and respiratory nuisance regressors in our GLM analysis.

We did not include a peripheral physiological measure to validate the success of conditioning. However, we carefully examined the behavioral and, most importantly, neural evidence with regard to conditioning success in a stepwise manner. We were able to demonstrate that our USs induced an arousal response for each threat type condition, and that the participants correctly anticipated the higher occurrence of the US during CS+ compared to CS− trials. At the neural level, we identified significant main effects of CS type (CS+, CS−) in our three regions of interest as well as on the whole brain level, with the latter results coinciding well with the whole-brain network that has been consistently identified in previous threat conditioning studies.

The USs used in our study differed in terms of sensory modality (audio-visual stimuli for predator attack and conspecific attack, painful electric shocks for pain) as well as behaviorally-reported arousal levels (higher for pain compared to the other two conditions, see behavioral results). We therefore designed our experiment carefully to circumvent this issue and focused our analysis on CS-related (instead of US-related) brain activity in the GLM analysis. We also carried out a control analysis on habituation effects on brain responses, since a different habituation to the three USs (e.g., a faster habituation to the audiovisual stimuli compared to electric shocks), could potentially influence our finding of threat-type dependent BOLD activations. We found no significant differential habituation effects for our three threat modalities (see also sections “2.9 Control analysis for habituation effects” and “3.2.5 Control analysis for habituation effects”). Nevertheless, we cannot completely rule out the possibility that some of the differences between pain and predator/conspecific attack could have been related to differences in stimulus intensity or modality.

A further limitation is the relatively limited variety of stimuli, since we only used three different stimuli for our three threat conditions. It would be interesting to include a variety of different threatening stimuli for each of the three conditions in future studies.

Lastly, our study included only female participants, whereas many rodent studies are performed in male populations. This design decision was based firstly on previously reported gender differences in emotion processing in general (122–124) and threat conditioning in particular (49, 125, 126) and secondly on the trade-off between increasing sample homogeneity (and thereby signal-to-noise ratio) and generalizability. Sex differences in learning and emotional memories have consistently been documented in rodents and humans (127). More specifically, in classical fear conditioning paradigms male rodents acquire CS-US associations faster than females (45). Defensive behaviors may also differ between male and female rodents, for instance freezing seems to be less common in female rats, whereas other defensive behaviors such as “darting” (rapid movements) has been found to be much more frequent in females (46, 47). Similarly, in humans larger conditioned responses during fear acquisition were found in men relative to women (48). Human fMRI studies on fear conditioning with painful stimuli have shown significantly greater BOLD-signal changes in the right amygdala, right rostral anterior cingulate (rACC) and dorsal anterior cingulate cortex (dACC) (49) and more insula activation (50) in women compared with men. In light of these differences, we made the decision to only include one gender in our study to increase sample homogeneity and thereby signal-to-noise ratio. This means, however, that our results are not generalizable to the male population.

Moreover, within a female sample, the level of sex hormones, in different phases of the menstrual cycle, or by the use of hormonal contraceptives, may influence fear conditioning (128, 129). More specifically, estradiol levels have been shown to influence the activation of brain regions underlying fear learning and extinction (129). We documented the phase of the menstrual cycle for each participant as well as whether they used hormonal contraceptives. Of the 21 naturally cycling females in our sample, only 1 single participant was in the second quarter of her cycle, in which estradiol levels are highest (130). This suggests that there was only a minimal effect of high estradiol levels on our results. However, we did not measure estradiol levels in our participants and cannot rule out the possibility that differences in estradiol levels may have influenced threat processing in our regions of interest. Future studies in relation to distinct threat type-dependent subcortical activations with male and female participants, taking hormonal levels into consideration, are needed.

### 4.8. Conclusion

In humans, different basic threats elicit distinct BOLD activations in the amygdala and the hypothalamus. More specifically, we find evidence for a dissociation between the threats of pain, conspecifics and predators in the human amygdala and hypothalamus. We believe that these findings are of interest as a first study investigating human subcortical neural correlates to distinct threat types and should be corroborated by further studies investigating the neurobiological basis of threat processing as well as studies including the male population and individuals with anxiety disorders, major depression and chronic pain.

## Data availability statement

The raw data supporting the conclusions of this article will be made available by the authors, without undue reservation.

## Ethics statement

This study was approved by the Ethics Committee at the Technical University of Munich. All participants gave their informed consent and received monetary compensation for their participation.

## Author contributions

CS, SMB, TB, DHA, and FB contributed to the conception and design of the study. DHA, MH, SMB, and TB performed the experiment and data collection. TB, DHA, SMB, and CS performed the statistical analysis. TB wrote the first draft of the manuscript. CS, SMB, and DHA wrote sections of the manuscript. All authors contributed to the manuscript revision, read, and approved the submitted version.
